# Evaluating the delay prior to primary care presentation in patients with lung cancer: a cohort study

**DOI:** 10.3399/BJGPO.2020.0130

**Published:** 2021-03-03

**Authors:** Jalpa Kotecha, Allan Clark, Matthew Burton, Wei Yee Chan, Di Menzies, Ulrike Dernedde, Rachel Banham, Andrew Wilson, William Craig Martin

**Affiliations:** 1 Department of Rheumatology, Epsom and St Helier University Hospitals NHS Trust, London, UK; 2 Norwich Medical School, University of East Anglia, Norwich, UK; 3 Department of Respiratory Medicine, Ipswich Hospital, Suffolk, UK; 4 Department of Haematology, University College London Hospitals NHS Foundation Trust, London, UK; 5 Department of Thoracic Surgery, Norfolk and Norwich University Hospital, Norwich, UK; 6 Oncology Department, James Paget University Hospital, Great Yarmouth, UK; 7 Community Nursing Office, Chedgrave, UK; 8 Oncology Department, Norfolk and Norwich University Hospital, Norwich, UK

**Keywords:** lung neoplasms, causes of delay, within-patient delay, primary health care, smokers

## Abstract

**Background:**

Little is known about 'within-patient delay', which is the time from first symptom of lung cancer to contacting primary care.

**Aim:**

Primary outcomes were length of within-patient delay and the proportion of total delay it represents. Secondary outcomes were factors causing delay and survival.

**Design & setting:**

A cohort study of newly diagnosed patients with lung cancer at two hospitals in Norfolk.

**Method:**

Patients completed questionnaires regarding onset of symptoms, whether they had delayed, and their reasons. GPs completed correlating questionnaires. Pathway times and other data were extracted from cancer registry and hospital records, and outcomes obtained prospectively. Factors causing delay were compared using ratios of geometric means.

**Results:**

In 379 patients, mean within-patient delay and pre-secondary care delay were 188.6 days and 241 days (61.4% and 78.5% of total delay, respectively). It was found that 38.8% of patients felt they had delayed. Patient-related causes of delay were denial (ratio of means [ROM] = 4.36; *P* = 0.002, 95% confidence interval [CI] = 1.71 to 11.1); anxiety (ROM = 3.36; *P* = 0.026; 95% CI = 1.16 to 9.76); non-recognition of symptoms (ROM = 2.80; *P* = 0.004; 95% CI = 1.41 to 5.59); and smoking (ROM = 1.76; *P* = 0.021; 95% CI = 1.09 to 2.86), respectively. These symptoms were associated with delay: finger swelling or discomfort (ROM = 2.72; *P* = 0.009, 95% CI = 1.29 to 5.74); cough (ROM = 2.53; *P*<0.001; 95% CI = 1.52 to 4.19); weight loss (ROM = 2.41; *P*<0.001; 95% CI = 1.49 to 3.88); weakness (ROM = 2.35; *P* = 0.001; 95% CI = 1.45 to 3.83); dyspnoea (ROM = 2.30; *P* = 0.001; 95% CI = 1.40 to 3.80); voice change (ROM = 1.90; *P* = 0.010; 95% CI = 1.17 to 3.10); and sputum (ROM = 1.66; **P** = 0.039; 95% CI = 1.03 to 2.67), respectively, also having more than five symptoms (compared with 1–3) (ROM = 3.69; *P*<0.001; 95% CI = 2.05 to 6.64). No overall relation between within-patient delay and survival was seen.

**Conclusion:**

Using smoking registers, awareness literature, and self-care manuals, primary care staff could liaise with people who have ever smoked regarding their symptoms to ensure early referral to secondary care.

## How this fits in

Patients with lung cancer present late, so there is much interest in reducing delay to treatment. The major phases of delay in patients’ pathways were studied, and patients who thought they had delayed were asked the reasons for their delay. The delay before the patient first contacts primary care is over 60% of total delay to treatment and its commonest cause is non-recognition of the symptoms. Public health measures and primary care networks need to ensure that at-risk people know the principal symptoms of lung cancer and, by using registers of people who have ever smoked, primary care staff could liaise with them regarding their symptoms to encourage early referral to computed tomography (CT) or secondary care.

## Introduction

Lung cancer is the commonest cause of cancer death worldwide.^1^ About seventy per cent of UK patients present in advanced stage,^2^ when curative surgery is not possible. The UK's overall 5-year survival of 10%^3^ is low by international standards.^4^ Recent approaches to improving results include: increasing awareness, screening, streamlining secondary care, and earlier identification in primary care.^5^

Improved awareness campaigns have shown short-term benefits,^6–8^ but are hard to sustain. Low-dose CT screening can improve mortality,^9,10^ but cost-effectiveness^11^ and implementation are problematic.^5,12^ Despite targets in secondary care,^13^ results remain poor. Interventions that target high-risk groups can improve respiratory consultation rates.^14,15^

Another approach is to focus on avoidable prolongation in the time from presentation to treatment (delay) and its causes. Cohort studies have used questionnaire models,^16,17^ case-notes review,^18,19^ and interview.^20,21^ The causes of delay include limited symptom awareness, anxiety, denial, and being too busy.^22–25^ Smokers are less likely than non-smokers to seek help for respiratory symptoms,^26^ and many of those at risk may normalise their symptoms.^27,28^

Delay worsens prognosis in patients treated with curative intent.^29,30^ However, in patients with lung cancer as a whole, the effect of delay is less clear, with some authors showing a longer time from presentation to treatment associated with better survival,^31,32^ others with worse.^18^ Possible reasons are that presenting symptoms can be non-specific,^17^ with uncertainty as to when the disease began, or that studies concentrating on major symptoms only^16,31^ may miss earlier ones.

Audits of delay often start from the first contact with primary care^33^ but ignore the earlier time from the first symptom. This within-patient delay^34^ may be prolonged,^20,27,35^ and contribute to poor outcomes. No study has compared all phases to treatment quantitatively.

There is, therefore, a need to quantify the length of within-patient delay, the proportion of delay it represents, the principal factors causing delay, and how all these affect survival.

## Method

### Design, setting, and governance

This cohort study involved questionnaires, tertiary care and cancer registry data, with outcomes obtained prospectively. Patients with a recent diagnosis of lung cancer were recruited from thoracic oncology clinics at the Norfolk and Norwich University Hospitals NHS Foundation Trust and James Paget University Hospitals NHS Foundation Trust between April 2008 and June 2012. The study was conducted according to good clinical practice.

### Patient recruitment

All patients with lung cancer were given an information pack and consent form with a freepost envelope, together with the patient questionnaire (PQ), by the doctor or research nurse at clinic. Patients were eligible if they provided written informed consent and were able to complete the 27-question questionnaire.

### Data collection

#### Patient data

The PQ asked about symptoms and events that led to the diagnosis, with dates of onset. This was in two sections.

The first section concerned symptoms and began: 'What was the first symptom that you feel was the beginning of this illness?' asking for the date or time in weeks since this developed. Exact dates were used if given, but where the patient gave only the month, the first of the month was used.^36^ Then followed 19 questions on specific symptoms, asking whether they had suffered these or not and, if so, the starting date or time in weeks. These symptoms were: dyspnoea, cough, sputum, purulence, haemoptysis, wheeze, chest pain, weight loss, anorexia, weakness, taste change, dry mouth, pyrexia, voice weakness and/or hoarseness (defined as voice change), finger swelling and/or discomfort (surrogate for clubbing), metastatic symptoms, dysphagia, neck lumps, and neck swelling. The questionnaire was self-administered and employed patient-friendly terms throughout. For example, rather than 'dysphagia', it asked 'have you had any trouble swallowing?' Finally, it asked about any other symptoms that they felt were part of the illness with dates. This type of questionnaire, asking whether the patient suffered a symptom and, if so, date of onset, has been validated in the C-SIM questionnaire.^37^

The second section asked whether they delayed in contacting their doctor and, if so, for what reason, when they saw them, the response, and how many times they saw them before being referred for a chest X-ray (CXR) or to secondary care. The questionnaire was reviewed early in the study to ensure it was well-understood and being completed satisfactorily, and continued, following correction of one spelling error.

#### Primary care data

GPs received a letter explaining the purposes of the study. This also asked them to complete a short questionnaire, asking the date first consulted about the symptoms that led to the lung cancer diagnosis, how many further consultations took place, dates of all consultations, and the date of referral to secondary care or for the CXR that led to secondary care referral.

### Outcomes

Primary outcomes were the length of within-patient delay and the proportion of total delay that it represents. Secondary outcomes were factors causing delay, and the relation between these and survival.

### Data handling and additional data retrieved

A research nurse recorded all questionnaire responses on a datasheet. Study researchers also retrieved data from hospital records and cancer registry, including: demographic patient data; age at diagnosis; date of diagnosis; postcode; index of multiple deprivation (IMD); tumour stage, site, and histology; treatment intent (radical or palliative); smoking data (dates of starting and quitting, pack-years); asbestos exposure, and patient status at close of study. Outcomes data were captured in October 2015, providing a minimum of 39 months follow-up. Dates needed to identify the phases in patients’ pathways (see below) were retrieved from cancer registry.

Symptoms consistent with known metastases, such as bone pain with a corresponding scan, were defined as 'metastatic symptoms', and analysed along with other symptoms.^18^ Non-specific symptoms, such as abdominal pain, were excluded from analysis.

Tumour, node, and metastasis (TNM) and stage-grouping followed the International Union against Cancer and American Joint Committee on Cancer seventh edition.^38^ All questionnaires, demographic data and key pathway dates were carefully checked. Following compilation of questionnaire data, the datasheet was anonymised. Date of diagnosis was defined as the date of biopsy that gave the histological diagnosis or, where there was no histology, date of radiological procedure that gave the diagnosis.^39^ Survival was measured from date of diagnosis until death, or the time of assessment, if still alive. The approaches to data collection, analysis, and reporting are consistent with the Aarhus statement on the design and reporting of studies on early cancer diagnosis.^39^

### Pathway phases

Five validated patient pathway phases, modified from Olesen *et al*,^20^ were defined as in Supplementary Table 1. This also shows the source of data; for example, whether from PQ or GP questionnaire (GPQ). Start of within-patient delay was taken as the earliest date given in response to all relevant questions.

The PQ and GPQ both recorded the date the patient first saw their GP, but in case of discrepancy, the GPQ date was used. Similarly the GPQ and cancer registry both recorded the date of referral to secondary care. Discrepancy was rare but, if this occurred, the cancer registry was used in preference.

### Analysis

Dividing patients into two groups based around median delay, it was calculated that 193 patients in each cohort would give 80% power to detect a survival difference between groups, with a hazard ratio of 1.337. Therefore, a sample size of 386 participants was chosen. This also permitted the use of up to 38 variables in the regression analysis.^40^ Survival within phases was also studied using subgroups according to length of delay. Descriptive analyses were reported on all measured variables for the whole group. These included demographic, patient, tumour and symptom details, additional data as above, and lengths of pathway phases (Supplementary Table 1).

Owing to the non-normal distribution, the model was based on the logarithm of delay.^41^ In order to aid interpretation, the resulting regression coefficients were exponentiated to give ratios of geometric means.^42^ For analysis of within-patient delay and survival, all variables above were included, also whether patients delayed and for what reason, each symptom, and the number of symptoms patients had. The final phase was not included in survival analysis since survival was measured from date of diagnosis.

These variables were assessed univariately using a regression model. All analyses were undertaken using Stata (version 14.1).

## Results

A total of 544 patients were recruited in order for 392 PQs to be returned. Thirteen were excluded — two non-lung primaries (thyroid and colorectal) and 11 questionnaires inadequately completed — leaving 379 for analysis. Of these, GPQs were completed in 266 (70.2%) patients.

Supplementary Table 2 shows demographic, smoking and tumour data, treatment intent, the number (%) of patients who delayed overall, commonest reasons for delaying, numbers (%) of patients with various numbers of symptoms and the current status.

Of the 363 (95.8%) patients answering the first question and also completing details on the symptoms section of the questionnaire, 137 (37.7%) mentioned symptoms that started before the date they gave as the beginning of the illness.

Mean within-patient delay was 188.6 days, 61.4% of total delay (median 84 days). Mean pre-secondary care delay was 241 days, 78.5% of total delay (median 142 days) (Supplementary Table 3).

Supplementary Table 4 shows how smoking, delaying, symptoms and number of symptoms all affected the length of delay in patients contacting primary care.

Age, sex, IMD, stage, histology, tumour site, and asbestos exposure were not found to influence within-patient delay.

As Supplementary Table 5 shows, survival was not altered by lengths of pathway phases, subgroups of within-patient delay, patients believing that they delayed, nor delay from various known causes. Stage and treatment intent strongly affected survival. Age, sex, IMD, and asbestos exposure did not affect survival.

Supplementary Table 6 shows, in a post-hoc analysis, how within-patient delay affected survival, after excluding patients who developed early metastatic symptoms.

## Discussion

### Summary

Within-patient delay, at 84 days median, is much the longest phase; over 60% of total mean delay. Likewise, pre-secondary care delay, median 142 days, is almost 80%. Current smokers delay longer. Denial, anxiety, and failure to recognise the symptoms are the most significant factors causing delay.

Seven symptoms are individually associated with longer within-patient delay. Also, the greater the number of symptoms a patient has, the longer is their delay. However, no overall relation was seen between within-patient delay and survival, nor between any pathway phase and survival.

### Strengths and limitations

The time of the first symptom was acccurately measured by asking closed questions on a large number of lung cancer-related symptoms, thereby accurately measuring within-patient delay. This length was compared with that of later pathway phases. Factors causing this delay have been identified, with their frequency, and their affect on delay and survival. The relation between number of symptoms and delay was studied. Finally, long-term survival data were obtained.

The seven symptoms associated with delay are intuitive. Dyspnoea, cough, and sputum are all common to benign disease;^17^ finger clubbing may not concern patients; weight loss and weakness may not initially cause anxiety; and patients with voice weakness or loss from lung cancer are often seen at ear, nose, and throat (ENT) before being referred to respiratory clinics, so delay can be expected with all these symptoms.

A limitation is that all symptoms data and dates of medical contact were obtained retrospectively by questionnaires, with the possibility of recall bias. However, symptom recognition from a checklist of symptoms (as on the questionnaire) has been shown to improve recall.^43^ Also a breast cancer study, which explored reasons for delay, achieved meaningful results despite patients being approached 3 months to 5 years after diagnosis.^44^ In any case, the primary care electronic records were cross-referenced and secondary care databases,^39^ and the outcomes data were obtained prospectively.

Since the patients were recruited from oncology, not all incident cases were captured. Had all patients receiving curative surgery been included, the relationship between within-patient delay and outcome might have been stronger, since patients in the early stage are likely to have had less delay. However, the % of staged patients in stage III-IV (86.3%) are similar to those of national data in England and Wales (72%–76%).^2^ It is, therefore, believed these findings apply to unselected patients with lung cancer presenting in primary care.

In completing the questionnaire, over a third of patients mentioned symptoms starting before the date they gave as the beginning of the illness (see Results). They, therefore, experienced symptoms before realising that they were ill and needing help. This illustrates the difficulty patients have in recognising symptoms. Other lung cancer researchers have asked separately about the start of the illness and dates of symptoms.^17^

Much of the data are over 5-years old. The delay in reporting relates to the authors' requiring long-term outcomes and having limited project staff. However, since the nature of lung cancer has not changed significantly, and outcomes remain poor, despite government targets,^13^ reducing within-patient delay is now an even more important aim.

### Comparison with existing literature

The results are consistent with an interview study that found 99 days median within-patient delay.^35^ The long delay before primary care is clinically important in view of published volume doubling times of 98 days in lung cancer.^45^ A case-control study found the incidence of symptoms was higher in cases than controls from as long as 2 years before diagnosis.^46^

The within-patient delay combines 'appraisal delay' (time to recognise one needs healthcare help) and 'help-seeking delay' (time to be seen).^47^ Since only three patients (0.8%) reported difficulty in making a GP appointment as a cause of delay (Supplementary Table 2), it is believed this is reasonable.

The study is the first to attempt accurately to compare pre-secondary with secondary care delay. The median primary care delay of 15 days is comparable to the recent national audit^33^ and the study's 2-week and 31-day waits are well within target. An early interview-based study of pre-secondary care delay found a median of 32.5 days, but possibly early symptoms were missed.^21^ The same study found that non-respiratory symptoms were associated with longer delay, but this has not been found in the present study.^21^

Of the causes of delay patients gave, denial and anxiety are the most significant. However, symptom non-recognition^23^ and smoking are more common. Patients in denial may not recognise, or admit to recognising, their symptoms, and those with anxiety may overlook symptoms, so these factors are not independent. Only 35% of smokers who had suffered cough or hoarseness over a 3-month period sought help, compared with 55% of ex- or never-smokers,^48^ which is consistent with the present study's finding that smokers delay longer. Longer pack-years of smoking are also associated with longer delay, as is chronic obstructive pulmonary disease (COPD).^35^

If patients who mentioned symptoms before the date given for the start of the illness are combined with the patients who declared symptom non-recognition as causing their delay, this shows that almost half the patients (46.7%) showed symptom non-recognition. In a recent UK population study, 38% of people could not recall any lung cancer symptoms.^49^ By contrast, international studies reported this percentage as 11.5%^43^ to 17%.^50^

#### Within-patient delay and survival

Within-patient delay is the longest phase, yet no relationship was seen between this and survival. Also, metastatic symptoms were not associated with delay (Supplementary Table 4), suggesting that a significant number of patients develop metastases early. In fact of 91 (24.0%) patients with metastatic symptoms, 34 (27.4%) developed these within 3 months of their first symptom.

After patients who developed metastatic symptoms within 3 months are excluded, in the remaining patients, those with a within-patient delay of 6–12 months had worse survival than those with short delay (Supplementary Table 6).

This suggests that two processes operate in lung cancer, both likely to worsen survival: metastases, which may occur early and unpredictably in lung cancer; and delay *to* treatment. This analysis would need to be repeated in larger series. However, this factor — the proportion of patients who develop early metastases — may explain contradictory findings in the literature between delay and survival, mentioned above.^18,31,32^

### Implications for research and practice

Since most delay occurs before any contact with primary care, this phase especially needs to be targeted. Action is needed in three areas: public health, primary care networks, and practices.

Symptom non-recognition remains common; therefore, increased awareness campaigns need to continue in public health and primary care networks. Current smokers or people who have ever smoked are identifiable through smoking registers available in primary care.^51^ A network nurse could liaise, by phone, text, or email, with these at-risk people, ensuring they receive proactive education, using self-help manuals,^15,52^ checking for lung cancer symptoms, and encouraging earlier referral for investigations or to secondary care. This is possible during the current COVID-19 crisis, and would require only modest funding.

In addition, every primary care consultation, COPD review, or health check in people who have ever smoked can be used for opportunistic questioning regarding lung cancer awareness and relevant symptoms.

The authors plan to report separately the study of symptoms in early detection of lung cancer in primary care.
